# A New Departure

**Published:** 1892-05-14

**Authors:** 


### THE INSTITUTIONAL WORKSHOP.

### ASYLUM ADMINISTRATION.

### A NEW DEPARTURE.

WE desire to enlist the interest and help of all our asylum readers in an effort now being put forth to open up more widely the range of topics bearing on the good work being done by our hospitals for the insane. We desire also to emphasise the importance of asylum service, not only as hospital nursing and care of the most arduous and beneficent character, but as a service too much neglected by the general public, and deserving even now what is a tardy recognition of its unobtrusive excellent] work. The public mind owes its misguided intelligence to sensational headings in the newspapers, and the far-fetched imaginations of the novel writer. Forgetting that "one swallow does not make a summer," it also forgets that one abuse does not prove a widespread evil, or one black sheep make grimy a whole flock. The public in its ignorance affects horror where there is nought to horrify. It therefore requires education, and with the help of our readers we hope to show asylums in a new light, to mark the remarkable strides of reform in all departments of the service, and to bring into relief the best and most pregnant ideas of the present day, bearing fruit in the management and treatment of the insane. By doing so we hope to give the work of mental nursing and care a more prominent place in the estimation of the public. The intelligent interest—and help if need be—of the British public is assured when once it grasps facts not fictions, and the elevation of asylum service then becomes a purpose of the first importance.

It is gratifying evidence of the enlightened views of asylum administrators, and the increasing attractions of this sphere of employment that the material of the service has immensely improved of late years ; the social grade is levelling upwards, and in respect to education, we find to-day many asylum nurses who may hold their own with the general run of hospital nurses. It cannot be denied, and it is much to be regretted, that hospital nurses are still apt to regard the two services as essentially incongruous the one with the other. But you cannot alter the protoplasm of human nature, and the only and obvious course for the asylum hospital service is to look up to a high standard and strive after it as consistently and successfully as has been done during the last few years. Probationer nurses come into asylums who would prefer the duties of a general hospital, but are under the specified age ; for the asylum regulation as to age is elastic. Thus, highly respectable, well educated girls find a labour field and raise the tone of the service.

The most important influence of all, however, has been the growing spread of education throughout the country. Unquestionably, the Education Acts have had a pronounced effect on the intelligence of this as well as other departments, and the candidate for asylum duty who can neither read nor write now is happily a *rara avis*. The old order of things found many advocates among the less enterprising superintendents, and there was a certain cogency in the argument they so often advanced, that education was often the veneer of an incapable, untrustworthy attendant and nurse ; but to say that because a person of education had applied for asylum service they must needs have been wrecks of some higher industrial grade, was not by any means necessarily true. The fact is that asylum service is much more attractive than it used to be, the amenities are much improved, and in many respects compensations that an hospital nurse would envy can be told off as equivalents for certain drawbacks, which make these institutions compare unfavourably with general hospitals.

A great deal is now being done to relieve the strain of work in asylum wards, and to make this vocation a branch of nursing in the best sense of the word. The efforts of individual superintendents have vied with each other in the direction of improved accommodation, remuneration, leave, and recreation ; and endeavours to train specially for this work by lectures, ward teaching, tutorial classes, and otherwise have culminated in a conjoint scheme of training and certificates under the auspices of the Medico-Psychological Association, which represents the branch of the medical profession engaged in asylum practice. The training scheme of the Medico-Psychological Association we will have occasion to explain in a future article; suffice it for the present to say that it marks an era in the history of asylums, as signifying the emancipation of asylum attendants and nurses from the old rut of "keeper" now becoming as obsolete as the "Mother Gamp" of the hospital.

As with all reforms, this change in our views of asylum function has not been effected without a persevering struggle ; but now that it has taken a start there will be no standing still. Evidence is accumulating on all sides of a widespread interest in this new movement, and as it must find expression somewhere through the agency of the press, we think no more appropriate medium can be found than the pages of THE HOSPITAL. The asylum page will be the mirror of all that is good and practical in the management and nursing of such institutions ; it will bring to a focus salient points in the everyday life and work of the wards, new devices for their comfort and cheerfulness, decorations, holiday festivities, and matters of interest or pressing importance to attendants and nurses themselves. We should like to offer, through the medium of THE HOSPITAL, facilities to all interested in this work, whether officially or otherwise, an opportunity of communicating to us any news of practical utility or any problems or difficulty of the service. In short, we ask our readers to help each other mutually by keeping us abreast of all the new ideas being advanced, of all improvements of whatsoever kind, and it will be our aim to make a judicious selection, and in a spirit of friendliness to help along the further elevation of the service.

We hope, from time to time, to publish brief descriptions of special features of new asylums, and also evidences of originality and enterprise in the work of the older asylums. We will also note what is done in furnishing, decorations, recreations, training, and examinations, and enlist special interest in the daily work of the wards, in observations of patients, and in the many side topics and incidents which can best be appreciated by those who are in daily personal contact with asylum duties. We trust, therefore, that the asylum page will embrace much. The more we bring the staff into touch with THE HOSPITAL, and the wide circle of its readers, the better for all concerned, and to do this broadly and effectually we desire hearty co-operation, and will gladly receive suggestions and promises of help from our readers.

